# Efficacy of Adjunctive Hyperbaric Oxygen Therapy in Osteoradionecrosis

**DOI:** 10.1089/biores.2018.0019

**Published:** 2018-10-08

**Authors:** Kamonwan Jenwitheesuk, Ajanee Mahakkanukrauh, Wiyada Punjaruk, Kriangsak Jenwitheesuk, Bowornsilp Chowchuen, Suthin Jinaporntham, Krittinant Uraiwan, Phasuk Limrattanapimpa

**Affiliations:** ^1^ Plastic and Reconstructive Surgery Unit, Department of Surgery, Faculty of Medicine, Khon Kaen University, Khon Kaen, Thailand.; ^2^ Division of Allergy-Immunology-Rheumatology, Department of Medicine, Faculty of Medicine, Khon Kaen University, Khon Kaen, Thailand.; ^3^ Department of Physiology, Faculty of Medicine, Khon Kaen University, Khon Kaen, Thailand.; ^4^ General Surgery Unit, Department of Surgery, Faculty of Medicine, Khon Kaen University, Khon Kaen, Thailand.; ^5^ Sleep Apnea Research Group, Research Center in Back, Neck and Other Joint Pain and Human Performance, Khon Kaen University, Khon Kaen, Thailand.; ^6^ Department of Oral and Maxillofacial Surgery, Faculty of Dentistry, Khon Kaen University, Khon Kaen, Thailand.; ^7^ Department of Dental, Srinagarind Hospital, Faculty of Medicine, Khon Kaen University, Khon Kaen, Thailand.; ^8^ Hospital Hyperbaric Oxygen Therapy Unit, Srinagarind Hospital, Faculty of Medicine, Khon Kaen University, Khon Kaen, Thailand.

**Keywords:** HBOT, hyperbaric oxygen therapy, osteoradionecrosis, prophylactic

## Abstract

Osteoradionecrosis (ORN) is a common consequence resulting from radiation in patients with cancer. Presently, hyperbaric oxygen therapy (HBOT) is proposed to have a role in improving wound healing in ORN patients. There is no strong scientific evidence to confirm the benefits of HBOT for treatment of ORN as an adjunctive treatment. This study aimed to determine the benefits of adjunctive treatment of HBOT in ORN. A retrospective study was conducted at the Srinagarind Hospital, the Faculty of Medicine, Khon Kaen University, Thailand, between 2011 and 2017. The patients diagnosed with ORN, who received adjunctive HBOT before the operation, were enrolled. Complete healing of wounds was the primary outcome. There were 84 ORN patients with a mean age of 58.78 years; 54.76% were male and 45.24% were female. HBOT had a role significant in improving wound healing of ORN patients with stages 1 and 2. Poisson regression analysis showed that stage 3 of ORN negatively correlated with the number of HBOT dives (*p* = 0.001, incidence rates ratio = 0.85). In conclusion, HBOT improved wound healing of ORN patients with stages 1 and 2. In addition, stage 2 of ORN patients significantly required the highest number of HBOT dives compared to other types of ORN to promote wound healing, whereas stage 3 patients, who underwent bone debridement combined with HBOT, initiated to success of treatment process and required a smaller number of dives.

## Introduction

Osteoradionecrosis (ORN) is a common consequence resulting from radiation in patients with cancer.^1,2^ The most commonly affected bone is the mandible.^3,4^ Patients with this malignancy generally receive conventional therapy, including surgery, chemotherapy, and radiation. Various forms of ionizing radiation have been used to treat cancer. In addition, ionizing radiation almost certainly has an unfavorable effect on soft and hard tissues.^5–8^ Consequently, affected soft tissues have been damaged and this has caused progressive endarteritis, hyalinization, and fibrosis. These lead to ischemia of affected tissues. Similarly, these adverse events have occurred in bone, resulting in destruction of local vascular systems and cellular components.^9,10^

In addition, histological findings of affected bone have presented decreased osteocytes and osteoblasts. Furthermore, periosteum is also affected and fibrosis may finally be formed and result in decreased bone remodeling. These changes progressively occur and may eventually lead to bone necrosis.^11^ Consequently, ORN patients often have poor treatment outcomes and high morbidity. Many malignant patients suffered from ORN after a complete course of cancer treatment.

There are several treatment modalities to improve wound healing in ORN patients. Both conservative and radical treatments are used to enhance the healing process of ORN.^12^ Presently, hyperbaric oxygen therapy (HBOT) is proposed to have a role in improving wound healing in ORN patients. HBOT is possibly recommended to use as adjunctive treatment with surgery to effectively promote the healing process in ORN patients.^13–15^ There is, however, no strong scientific evidence to confirm the benefits of HBOT for treatment of ORN as an adjunctive prophylactic treatment. Therefore, further studies about the effects of HBOT are still required to support the advantages of using HBOT in ORN patients.

## Materials and Methods

The retrospective study design was to determine the influence of HBOT in ORN patients. This study was conducted from 2011 to 2017 at Srinagarind Hospital, the Faculty of Medicine, Khon Kaen University, Khon Kaen, Thailand. There were 84 patients included in this study. The inclusion criteria for this study were all stages of ORN patients, who received HBOT as adjunctive prophylactic treatment before and after the procedure.

All patients had the evidence of previous radiotherapy for head and neck cancer. Criteria for staging were as follows: stage 1, determined as asymptomatic exposed bone; stage 2 was exposed bone with associated symptoms such as pain, soft tissue inflammation, or even infection; and stage 3, similar symptoms and signs as stage 2 and combined with sequestrum, pathological fracture, or orocutaneous fistula. Medical history, physical examination, and plain X-ray were performed in all patients with noncomplicated disease; those patients with severe presentation required a computed tomography, especially in stage 3.

The primary outcome of the study was wound healing, which was defined by complete soft tissue coverage to bone without any infection, inflammatory process, or fistula. The evaluation of wound healing was documented after a complete course of treatment and at least 6 months to examine for the disease-free state.

All demographic data and clinical data of patients were recorded, including age, sex, types of primary cancer, stages of ORN, surgical procedures, HBO therapy (total oxygen time and number of dives), and outcome of wound healing. For the protocol of treatment, patients were treated daily with HBO for 20 dives before they had surgery, and 10 more HBO dives were given to patients after the surgical procedure. Therefore, there were a total 30 dives of HBO. If, however, the wound did not heal, an extension of treatment would be considered. In addition, patients were treated with HBO daily at 2.4 atmospheres absolute for 90 min in a monoplace chamber. The surgical procedure for stages 1 and 2 ORN patients was dental extraction, while stage 3 was bone debridement.

### Statistical analysis

For the demographic data of patients, frequencies of data are presented for the categorical data, and mean (standard deviation) or median (interquartile range) are shown for continuous data. Normal distribution and equality of variances were analyzed for all continuous variables.

The generalized linear model (GLM) was used to determine the correlation between important variables and treatment outcomes of HBOT. This model was used to determine significant variables, which were independently associated with HBOT. GLM was also performed with the Poisson family and log for link functions analysis. A Poisson regression was used to correct the overdispersion of GLM using the Bootstrap test because HBOT count is used in data. HBOT outcomes were adjusted for age, sex, types of primary cancer, and stages of ORN.

This study was approved by the Khon Kaen University Ethics Committee for Human Research (HE611060).

## Results

There were 84 ORN patients with ORN included in this study. The mean age of patients included in this study was 58.78 years (Table 1). Both sexes were included nearly equally (54.76% were male and 45.24% were female). In addition, the mean of total number of HBOT dives was 29.83. The majority of patients had primary cancer of oral cavity (70.24%), followed by nasopharynx (23.81%) and neck cancer (5.95%). In addition, most patients were in stage 1 for 63.10%, only 8.33% of patients were in stage 2, and in stage 3, there were 28.57% of patients.

**Table 1.** **Baseline Characteristics of Patients with Osteoradionecrosis, Who Received a Hyperbaric Oxygen Therapy as an Adjunctive Prophylactic Treatment**

Variable	Mean/no.	SD/%
Age	58.78	11.76
Male	46	54.76
Total number of dives	29.83	6.14
Primary cancer		
Nasopharynx	20	23.81
Oral	59	70.24
Neck	5	5.95
Stage of ORN		
1	53	63.10
2	7	8.33
3	24	28.57

ORN, osteoradionecrosis; SD, standard deviation.

Univariate analysis was performed and the results revealed that there was only one significant variable that was associated with the number of HBOT dives, resulting in healed wounds. These data that included stages of disease are presented in Table 3. Poison regression analysis showed that stage 3 of ORN was negatively correlated with the number of HBOT dives (*p* < 0.01, incidence rates ratio [IRR] = 0.85; Table 2). Multivariate analysis showed the same results, which successfully promoted wound healing of ORN patients as presented in Table 3. Only stage 3 of ORN significantly had a negative correlation with the number of HBOT dives (*p* = 0.001) using Poisson regressive analysis (incidence rates ratio [IRR] = 0.856).

**Table 2.** **Univariate Analysis of All Variables Influencing Treatment Outcomes in Patients with Osteoradionecrosis, Who Received a Hyperbaric Oxygen Therapy as an Adjunctive Prophylactic Treatment**

Variables	IRR	95% CI	*p*
Stage			
1	Ref.		
2	1.10	0.96–1.26	0.13
3	0.85	0.77–0.93	<0.01
Male	0.96	0.89–1.04	0.36
Age	1.01	0.99–1.01	0.23
Nasopharynx	0.98	0.90–1.08	0.79
Oral	1.01	0.92–1.09	0.83
Neck	1.01	0.85–1.18	0.94

CI, confidence interval; IRR, incidence rates ratio.

**Table 3.** **Multivariate Analysis of Important Variables Influencing Treatment Outcomes in Patients with Osteoradionecrosis, Who Received a Hyperbaric Oxygen Therapy as an Adjunctive Prophylactic Treatment**

Variables	IRR	95% CI	*p*
Age	1.001	0.998–1.004	0.576
Stage			
2	1.122	0.977–1.288	0.104
3	0.856	0.780–0.939	0.001
Female	0.939	0.867–1.018	0.125

## Discussion

ORN is a complication of radiation therapy in head and neck cancer.^16^ ORN, when it happens, generates a problem for a complex therapeutic process such as free tissue transfer.^17,18^ The treatment outcomes are often not as expected and may cause an unfavorable result.^19^ Some procedures such as tooth extractions or even debridement can cause a progression of ORN.^20,21^ It is necessary for the physician to consider prevention protocols before performing jaw procedures.^22^ Presently, several previous studies proposed that HBOT has a significant role in the management of ORN patients.^23–25^

ORN patients included in this study were treated with HBOT as adjunctive and prophylactic treatments. This study determined the treatment outcome of HBOT. Poisson regression analysis showed that only stage 3 of ORN significantly had a negative association with the number of HBOT dives, which led to a healed wound analyzed using both univariate (IRR = 0.85) and multivariate analysis (IRR = 0.856). Significantly, stage 3 required a lower number of HBOT compared to stage 2, as patients with stage 3 had already undergone bone debridement, while bone debridement had not been performed in stage 2 of disease. Basically, stage 3 of ORN has severe bone necrosis and is of worse severity than stage 2. Consequently, ORN patients with stage 3 normally received early bone debridement or bone resection.

Therefore, HBOT did not play a major role in promoting wound healing because problematic bone was already removed. Hence, a smaller number of HBOT dives were required for stage 3 by an adjunctive treatment of ORN patients to enhance wound healing compared to stage 2 of disease. Compared to other studies, these results were consistent with previous studies and it revealed that a high success rate of treatment was found in overt ORN patients and HBOT significantly provided an advantage in prophylactic use in patients who needed dental extraction and had a risk of developing ORN.^26,27^ Shaw suggested to prevent unfavorable outcomes of ORN with HBOT before any combination of surgery, which is similar to our study.^28^

One study, however, revealed that HBOT did not provide any benefit for overt ORN patients and it did not recommend using HBOT for these patients.^29^ Thus, there is still a controversy about using HBOT in overt ORN. In addition, stage 1 of ORN also required a small number of HBOT dives because the lesion was in an early stage and less severe. Vudiniabola also reported that HBOT was the effective treatment in stage 1.^30^ Therefore, ORN patients with stage 1 could simply be healed when they were treated with HBO with a small number of dives.

The most appropriate treatment for stage 2 of ORN patients having less bone necrosis compared to stage 3 was often difficult to plan treatment because the lesion is not bad enough to have early surgery, which includes bone debridement or bone resection. Therefore, these patients can likely be cured with conservative treatment. Stage 2 of ORN patients essentially required a large number of HBOT dives to effectively enhance wound healing. These results, also previously reported by other studies, show that the success rate of HBOT in this stage was not appreciated like in stage 1.^27,30^

Conversely, if ORN patients with stage 2 had early bone debridement or bone resection and then were treated with HBO, patients seemed to have a good advantage compared to delayed surgery, while waiting for the treatment outcome of HBO. Therefore, surgical therapy should be considered early for ORN patients with stages 2 and 3 to effectively promote wound healing and decrease the length of hospital stay for HBOT. This hypothesis can be tested by more studies in the future, with the assessment of cost-effectiveness and quality of life.

## Conclusion

HBOT had a significant role in improving wound healing of ORN patients in stages 1 and 2. In addition, patients in stage 2 of ORN significantly required the highest number of HBOT dives compared to other types of ORN to promote wound healing, whereas patients in stage 3, who underwent bone debridement, initiated to success of treatment process. Bone debridement essentially enhances wound healing in ORN patients with a severe ORN status, resulting in using a smaller number of HBOT dives.

## Abbreviations Used

CI: confidence interval
GLM: generalized linear model
HBOT: hyperbaric oxygen therapy
IRR: incidence rates ratio
ORN: osteoradionecrosis
SD: standard deviation
